# Heterologous Expression of Ralp3 in *Streptococcus pyogenes* M2 and M6 Strains Affects the Virulence Characteristics

**DOI:** 10.1371/journal.pone.0055109

**Published:** 2013-02-12

**Authors:** Nikolai Siemens, Bernd Kreikemeyer

**Affiliations:** Institute of Medical Microbiology, Virology, and Hygiene, Rostock University Hospital, University of Rostock, Rostock, Germany; University of South Dakota, United States of America

## Abstract

**Background:**

Ralp3 is a transcriptional regulator present in a serotype specific fashion on the chromosome of the human pathogen *Streptococcus pyogenes* (group A streptococci, GAS). In serotypes harbouring the *ralp3* gene either positive or negative effects on important metabolic and virulence genes involved in colonization and immune evasion in the human host were observed. A previous study revealed that deletion of *ralp3* in a GAS M49 serotype significantly attenuated many virulence traits and caused metabolic disadvantages. This leads to two questions: (i) which kind of consequences could Ralp3 expression have in GAS serotypes naturally lacking this gene, and (ii) is Ralp3 actively lost during evolution in these serotypes.

**Methodology/Principal Findings:**

We investigated the role of Ralp3 in GAS M2 and M6 pathogenesis. Both serotypes lack *ralp3* on their chromosome. The heterologous expression of *ralp3* in both serotypes resulted in reduced attachment to and internalization into the majority of tested epithelial cells. Both *ralp3* expression strains showed a decreased ability to survive in human blood and exclusively M2::*ralp3* showed decreased survival in human serum. Both mutants secreted more active SpeB in the supernatant, resulting in a higher activity compared to wild type strains. The respective M2 and M6 wild type strains outcompeted the *ralp3* expression strains in direct metabolic competition assays. The phenotypic changes observed in the M2:*ralp3* and M6:*ralp3* were verified on the transcriptional level. Consistent with the virulence data, tested genes showed transcript level changes in the same direction.

**Conclusions/Significance:**

Together these data suggest that Ralp3 can take over transcriptional control of virulence genes in serotypes lacking the *ralp3* gene. Those serotypes most likely lost Ralp3 during evolution since obviously expression of this gene is disadvantageous for metabolism and pathogenesis.

## Introduction

*Streptococcus pyogenes* (group A streptococci, GAS) is an important and exclusively human pathogen capable of causing a wide variety of infections in its human host. Most frequently infections comprise mild superficial diseases of the throat and skin and infections of deep-seated connective tissues or septicaemia [1], [2]. Although the molecular mechanisms of GAS pathogenesis are not fully understood, one of the best-characterized mechanisms at an initial infection step is the specific and non specific binding of GAS to epithelial cells [3]. This attachment relies on interactions of the GAS surface with host cell surface or plasma molecules. Fibronectin-binding surface proteins of GAS have been reported to be major molecules for eukaryotic cell adhesion and internalization [4]–[7]. GAS also use host plasma molecules like plasminogen for adherence to, internalization into, and transmigration through eukaryotic cells [8]–[11].

The targeting of different tissues and usage of various ways to attack their host cells during the different stages of GAS infection requires a balanced activity of several regulators and two component systems [12]. The longest known and best characterized virulence regulators are Mga, RofA/Nra, and Rgg/RopB [13]–[16]. It has been demonstrated that the activity of these regulators affects the expression of various virulence factors, in most instances in a serotype dependent manner.

Transcriptome analysis of a serotype M49 GAS strain and its isogenic Nra knock out mutant discovered the transcriptional control of an additional stand-alone regulator, Ralp3 (RofA-like protein regulator type 3 [14]). *ralp3* homologous genes were exclusively found in serotypes M1, M4, M12, M28 and M49. *ralp3* is linked with a gene encoding Epf (extracellular protein factor). In a serotype specific mode, this *ralp3 epf* gene block is integrated into the *eno sagA* gene block encoding for a plasminogen binding enolase and a streptolysin S precursor. The complete *eno ralp3 epf sagA* region was called ERES pathogenicity island [14]. Deletion of Ralp3 in GAS M49 reduced the adherence to and internalization into human keratinocytes, severely attenuated survival of the mutant in human blood and serum, reduced the plasminogen-binding capacity, decreased the secretion of the important virulence factor SpeB, and finally downregulated expression of genes encoding proteins important for metabolism of fructose and lactose [17]. Together that study suggested a rather beneficial activity of Ralp3 expression for many virulence attributes and also metabolism of the GAS M49 serotype.

The question remained if presence of the *ralp3* gene in GAS M1, M4, M12, M28 and M49 is rather a gain of function emerged during pathogen evolution, or if lack of this gene in the *ralp3-*negative serotypes can be considered a loss of function? To approach this question, in this study we investigated if heterologous expression of the *ralp3-*gene in *ralp3*-negative GAS serotypes allowed transcriptional control of Ralp3 on genes encoding proteins involved in virulence and metabolism, and if this is beneficial or of disadvantage for the in vitro virulence phenotype of such strains.

## Materials and Methods

### Bacterial Strains, Eukaryotic Cells, and Culture Conditions

GAS serotype M49 strain 591 is a skin isolate provided from R. Lütticken (Aachen, Germany). The M2 and M6 serotype GAS wild type strains are clinical isolates obtained from the collection of the Centre of Epidemiology and Microbiology, National Institute of Public Health, Prague, Czech Republic. GAS M49::mock with an empty pAT29 plasmid was used for the direct competition experiments. *E. coli* DH5α was used as a host for pAT18_*ralp3* plasmid and was cultured in Luria-Bertani medium at 37°C with agitation. The GAS wild type strains and the mutants were cultured in Todd-Hewitt broth (Invitrogen) supplemented with 0.5% (w/v) yeast extract (THY; Invitrogen) at 37°C under a 5% CO_2_–20% O_2_ atmosphere. For selection of the mutants, antibiotics were added to the media at following concentrations: erythromycin 5 µg ml^−1^ for GAS and 300 µg ml^−1^ for *E. coli*.

The human epithelial cell lines HaCaT (Deutsches Krebsforschungszentrum (DKFZ), Heidelberg), HEp-2 (ATCC CCL23), and Detroit 562 (ATCC CCL-138) were used for a standard adherence and internalization assay. The keratinocytes were maintained in Dulbecco modified Eagle medium (DMEM; Gibco) GlutaMAX™-I, 10% (v/v) fetal bovine serum (FBS; Gibco). HEp-2 cells were maintained in DMEM, 2 mM L-glutamine, 10% (v/v) FBS, and Detroit 562 cells were maintained in DMEM:F12, 2 mM L-glutamine 10% (v/v) FBS.

### Construction of Recombinant Vectors and GAS Strains

The construction of a pAT18_*ralp3* vector was previously published [14]. Transformation of GAS M2 and M6 was performed as described previously [18]. The generated *ralp3* expression strains were designated: M2::*ralp3* and M6::*ralp3*. To confirm the transcription of *ralp3*, total RNA from GAS wild type and mutant strains from 20 ml of culture at the transition phase of growth was isolated with the FastRNA Pro Kit (MP Biomedicals) according to the manufacturer guidelines. A probe for Northern hybridization was generated by PCR using the following primer: *ralp3_*probeF (5′-GAGTTCAGAATTCTTACACCTTCATCTAGAAACC-3′) and *ralp3_*probeR (5′-CTTTATGTCGACAGGAGAAGACAGGTTCTTGTG-3′).

### Quantitative Real Time PCR (qRT-PCR)

For qRT-PCR synthesis of cDNA was performed using the Superscript First-Strand Synthesis System for RT-PCR (Invitrogen). Primer sets for selected genes are listed in Table 1. The real time-PCR amplification was performed with SYBR Green (Fermentas) using an ABI PRISM 7000 Sequence Detection system (Applied Biosystems). The level of DNA gyrase subunit A gene (*gyrA*) transcription was used for normalization. For data comparison, the normalized values were calculated by Log_2_ expression ratio.

**Table 1:** Primers used in qRT-PCR experiments in this study.

Gene	Primer	Sequence (5′-3′)
*fruK*	fruK-for	CGTGACCTTAAACCCTTCTATTG
	fruK-rev	CCTCCAGCAAACTTATCATCACT
*lacA1*	lacA-for	TTAGGCCATTTTATTGAGCATGTCA
	lacA-rev	TCCAGAATTAGCGAAGAATATCGTC
*lacA2*	lacA2-for	TTAGCACATTTTATTAAGCATGTCGAC
	lacA2-rev	TCCAGGAATTGCAAAGAACATTATC
*emm*	emm-for	AACGCTAAAGTAGCCCCACAAG
	emm-rev	TCGCCTGTTGACGGTAACG
*mga*	mga-for	TTCACAGATAACAACGTTATGGTCAA
	mga-rev	TTCTGGTTTTTGTACCTCTTTGTCACT
*gyrA*	gyrA-for	CGACTTGTCTGACGCCAAA
	gyrA-rev	TTATCACGTTCCAAACCAGTCAA
*speB*	speB-for	CCA CCC CAA CCC CAG TTA A
	speB-rev	GGCGGACATGCCTTTGTTAT
*ska*	ska-for	GCTGACAAAGATGATTCGGTAACC
	ska-rev	GCA CATGCCCACTTAGCAAA
*rofA*	rofA-for	CCCGCTTGTTTGGTTTGAGT
	rofA-rev	GGTGCAAGGCTAAGATGGTTTT
*eno*	eno-for	GCTGACATCGCAGTTGCAA
	eno-rev	CAATACGGTCTGTACGTGACAATG
*sagA*	sagA-for	TGCTGCTGTACTACTTGTTGCTTCT
	sagA-rev	GCTAAATAGATTATTTACCTGGCGTATAACT

### Direct Competition Experiments

For the direct competition experiments of wild type and mutant strains equal CFU of each strain were inoculated into THY, CDM-lactose, and CDM-fructose. At time points 0, 4, and 8 h postinoculation, the colony forming units (CFU) for both strains were determined from the mixtures by plating serial dilutions on agar plates. The antibiotic resistance of the mutant allowed a numerical discrimination and evaluation. Wild type and *ralp3* expression strain single cultures grown under the same conditions served as controls.

### Eukaryotic Cell Adherence and Internalization

Adherence to and internalization into epithelial cells was quantified using the antibiotic protection assay [19]. 24-well plates were inoculated with 2.5×10^5^ cells per well in DMEM medium without antibiotics. Cells were allowed to grow to confluence. For the assay, cells were washed with DMEM, and infected with GAS wild type and M2::*ralp3*/M6::*ralp3* strains at a multiplicity of infection of 1∶10 in DMEM. Two hours after infection, cells were washed extensively with PBS, detached from the wells by trypsin treatment, and lysed with sterile distilled water. The viable counts of GAS (CFU) released from the lysed cells were determined by serial dilution in PBS and plating on THY agar. For the assessment of bacterial internalization, 2 h after infection the cells were washed with PBS and incubated with DMEM supplemented with penicillin (50 U/ml) and streptomycin (5 mg ml^−1^) for additional 2 h. Subsequently, the cells were washed and lysed and the GAS viable counts were determined as described above.

### GAS Survival in Different Human Media

The survival assay was performed as described by Nakata et al. [20]. Briefly, overnight cultures of the wild type and *ralp3* expression strains were inoculated into fresh medium and grown to exponential growth phase. Bacteria were harvested by centrifugation and suspended and diluted in PBS. 20 µl of each suspension were inoculated into 480 µl of heparinized blood and blood serum to a bacterial count of 5–10×10^3^ CFU ml^−1^. After 3 h of incubation at 37°C with rotation, the CFU were determined by plating and compared to the inoculum.

### Cysteine Protease Activity Assay

The SpeB protease activity in GAS culture supernatants was determined by using the method adapted from Hytonen et al. [21]. Briefly, the bacteria were grown in THY medium to the transition phase, harvested by centrifugation, and the supernatants were collected. 100 µl of the filtered (0.22 µm pore size) supernatants were incubated with dithiothreitol (DTT; 1 mM) for 30 min at 37°C to activate the enzyme. Then *n*-benzoyl-proline-phenylalanine-arginine-*p*-nitroanilide hydrochloride (1 mM) and phosphate buffer (pH 6; 5 mM) were added to the activated supernatants. The change in absorbance at 405 nm was detected in a spectrophotometer. E64-cystein protease inhibitor was tested in parallel assays to confirm the specificity of the test.

### Streptokinase Activity Assay

The assay was performed by using a method adapted from Ringdahl et al. [22]. Briefly, the bacteria were grown in THY medium to the transition phase, harvested by centrifugation, and the supernatants were collected. 100 µl of the filtered (0.22 µm pore size) supernatants were mixed with plasminogen (1 µM) and incubated in 100 µl of plasmin substrate solution, prepared by mixing 2 volumes of chromogenic substrate H-D-Val-Leu-Lys-ρ-nitroanilide (Sigma) stock solution (5 mg ml^−1^) with 3 volumes of 32 mM Tris/1.77 M NaCl (pH 7.5), for 15 min at 37°C, followed by absorbance measurement at 405 nm. THY mixed with plasmin (1 µM) was used as a positive control. All Ska activities were related to the activity of the plasmin control.

## Results

### Substrate Utilization by GAS M2 and M6

**Figure 1:**
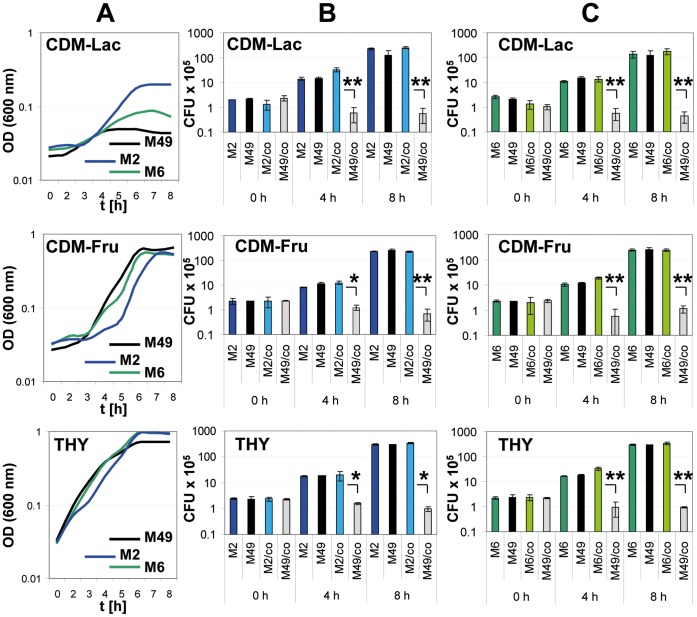
Growth and direct competition profile of GAS Serotypes M2, M6, and M49 wild type strains. (A) Growth of GAS Serotypes M2, M6, and M49 wild type strains in THY and CDM supplemented with lactose (Lac) and fructose (Fru). Determination of GAS M2/M49 (B) and M6/M49 (C) CFU during the growth on CDM-Lac, CDM-Fru, and THY. M2, M6, and M49: wild type control; M2/co - M49/co and M6/co - M49/co: both strains in a direct competition. The level of significance was calculated by t-Test (n = 3; *p<0.05; **p<0.01).

Since the regulatory network of GAS M49 was published, it is known that Ralp3 regulates genes with metabolic function, especially both *lac* and *fru* operons [17]. To compare the ability of different serotypes to utilize sugars, growth experiments in CDM batch cultures with lactose and fructose as sole carbon source were performed. As shown in Fig. 1A all tested serotypes are able to utilize both sugars. On CDM-lactose M2 and M6 reached higher ODs than GAS M49. On CDM-fructose slight differences between the serotypes were noted in the exponential growth phase. No difference in growth behavior on THY complex medium was observed. In order to analyze if the *ralp3-*gene negative serotypes M2 and M6 have any selective growth advantage in direct competition, we performed growth experiments with both strains (M2 vs. M49 and M6 vs. M49) at equal starting CFU in the same culture. As shown in Fig. 1B and C at both tested C-sources and on THY the *ralp3*-negative GAS serotypes M2 and M6 outcompeted the growth of M49. At time point 0 h post-inoculation equal CFU were recovered from the mixed cultures. During growth in all tested media the GAS serotypes M2 and/or M6 achieved a higher CFU number at exponential (4 h) and stationary (8 h) growth phase.

**Figure 2:**
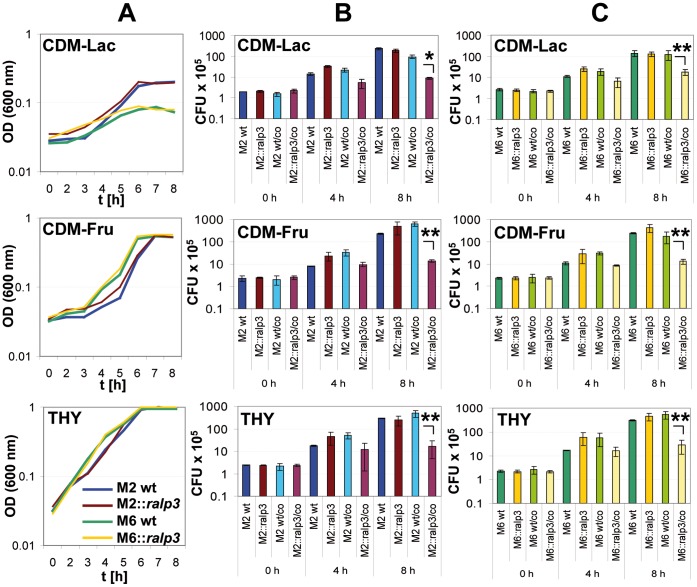
Growth and direct competition profile of GAS M2, M6 wild type and corresponding M2::*ralp3* and M6::*ralp3* strains. (A) Growth of GAS M2, M6 wild type and corresponding M2::*ralp3* and M6::*ralp3* strains in CDM-Lac, CDM-Fru, and THY. (B) Determination of the CFU from GAS M2 (B) and M6 (C) during the growth on CDM-Lac, CDM-Fru, and THY. wt: wild type control; ::*ralp3*: *ralp3* expression control; wt/co and ::*ralp3*/co: both strains in a direct competition. The level of significance was calculated by t-Test (n = 3; *p<0.05; **p<0.01).

Next we tested the ability of M2::*ralp3* and M6::*ralp3* strains to utilize the same sugars. No differences between wild type and mutant strains were observed (Fig. 2A). To analyze whether the wild type or *ralp3* expression strains have any selective growth advantages in direct competition, we determined the CFU of both strains in the same culture. As shown in Fig. 2B and C, at the time point 0 h post inoculation and 4 h (exponential growth phase) equal numbers of CFU were recovered from M2 and M6 mixed cultures. During growth from late exponential until stationary phase, both wild types suppressed the growth of the *ralp3* expression strains in all tested media, documented by significantly higher CFU numbers of wild type strains. From a metabolic point of view the expression of Ralp3 in these serotypes seems to be a growth disadvantage.

### Ralp3 Suppressed the Ability of the Bacteria to Adhere to and Internalize into Human Epithelial Cells

**Figure 3:**
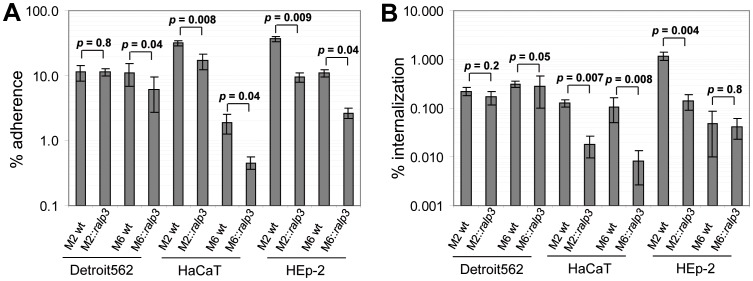
2 h Infection of human epithelial cells. (A) Adherence to and internalization (B) into human epithelial cells by GAS M2 wild type, M6 wild type, M2::*ralp3*, and M6::*ralp3* strains. The data represent mean values ± standard deviations from four independent biological experiments. The level of significance was calculated by U-test.

In the next set of experiments we studied the effect of Ralp3 on the phenotype of adherence to and internalization into human epithelial cells, a relevant step during GAS laryngeal, pharyngeal, and skin infections. Detroit562, HEp-2, and HaCaT cells were used as target cells, respectively, and were infected with GAS Serotypes M2, M6 wild type strains in comparison to M2::*ralp3*, M6::*ralp3* strains. Figure 3 summarizes the results of these experiments. Heterologous expression of *ralp3* leads to decreased ability of GAS serotypes M2 and M6 to adhere to and internalize into HEp-2 and HaCaT cells. The adherence to and internalization of GAS M2::*ralp3* into Dertoit562 cells was not significantly attenuated. Moreover, GAS M6::*ralp3* and wild type strains showed only insignificantly different internalization into Detroit562 and HaCaT cells. Together, these results again establish a negative effect of Ralp3 expression on important GAS M2 and M6 virulence characteristics.

### GAS Survival in Different Media

**Figure 4:**
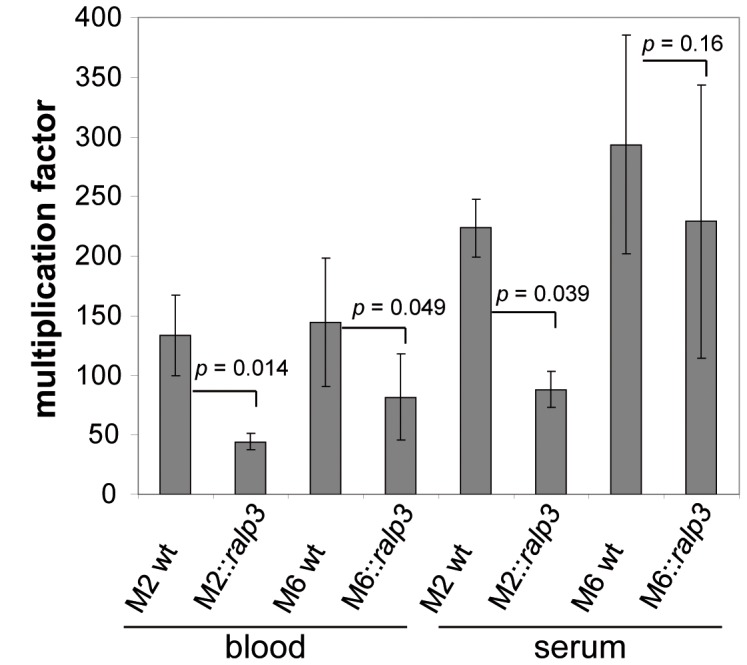
Survival of wild type and *ralp3* expression strains in human whole blood and human serum. The data of the survival assays represent the mean values ± standard deviations from four independent biological experiments. The number of surviving CFU was determined by plating serial dilutions and subsequent counting. The *y* axis shows the resulting multiplication factor for each strain calculated from the percentage of surviving CFU related to the inoculum. The level of significance was calculated by U-test.

As already summarized in Fig. 2A, no significant differences were observed between the strains if grown in THY medium. To extend this investigation to more *in vivo*-like conditions, we subsequently determined the ability of wild type and *ralp3* expression strains to survive in different human media as described by Nakata et al. [20]. As demonstrated in Fig. 4 both heterologous Ralp3 expression mutants showed a significantly decreased ability to survive in human blood. In addition, GAS strain M2::*ralp3* was attenuated in its survival rate in human serum. The expression of *ralp3* in GAS serotype M6 did not influenced the ability of GAS M6 to survive in human serum. Again these results established a potential link between loss of the *ralp3*-gene and an increased virulence of the M2 and M6 GAS wild type strains.

### Ska and SpeB Activity in GAS Culture Supernatants

**Figure 5:**
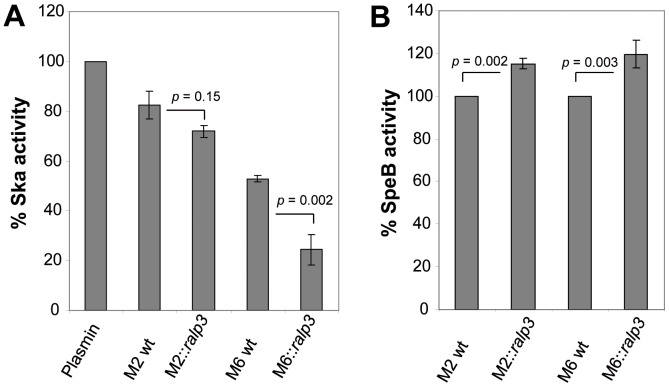
The activity of secreted GAS virulence factors in culture supernatants. (A) Ska activity of wild type and *ralp3* expression strains was measured by conversion of plasmin substrate solution at OD_405 nm_. All Ska activities were correlated to the activity of plasmin, which was set to 100%. (B) SpeB activity of culture supernantants. The supernantants were activated by DTT, and proteolytic cleavage of the substrate was measured at OD_405 nm_. The level of the significance was calculated by U-test (n = 4).

The activity of secreted GAS virulence factors was examined in culture supernatants of the wild type and expression strains. As factors of major interest, GAS streptokinase (Ska) and cysteine protease (SpeB) were analysed. Functional analysis of the plasminogen activator Ska in the supernatant of the M6::*ralp3* revealed significantly lower activity than parallel samples from GAS M6 wild type (Fig. 5A). The additional heterologous expression of *ralp3* in GAS M2 background did not affect the activity of Ska to reach significant levels.

Subsequently, we compared SpeB activity of the GAS M2 and M6 wild type with M2::*ralp3* and M6*::ralp3* strains. The SpeB activity of the wild type strains was set 100% and the measurements of the supernatants of M2::*ralp3*/M6::*ralp3* were related to the wild type stains. As shown in Figure 5B, both *ralp3* expression strains showed significantly increased SpeB activities resulting from a likely increased secretion of this protein in the environment. These results lead to the question if Ralp3 expression operates on the level of transcription in controlling genes encoding Ska and SpeB.

### Transcription Analysis of Relevant Virulence Factor Encoding Genes

The phenotypic changes observed in M2:*ralp3* and M6:*ralp3* could result from effects of Ralp3 on virulence factor transcription. Therefore, qRT-PCR analysis with RNA from wild type and *ralp3* expression strains was performed. The transcript level profile was determined by log_2_ expression ratio. Genes were considered significantly different when the log_2_ ratio of the fold change was ≥1.0 or ≤ −1.0 (Table 2). Both mutants displayed elevated levels of *speB* and *lacA1* transcription. Additionally, the transcription of *rofA* and *sagA* were significantly induced in M2::*ralp3* strain. The transcription of the *emm* gene was significantly reduced in both *ralp3* expression strains. A decrease in *mga* and *rofA* transcription in GAS M6::*ralp3* background was also detected. No significant changes of *eno*, *lacA2*, and *fruK* mRNA levels were observed in both expression strains.

**Table 2:** Genes down and up regulated by *ralp3* expression in GAS M2 or M6.

Gene	M2*::ralp3*	M6*::ralp3*
***mga***	0.76±0.73	**−1.66±0.25**
***emm***	**−1.45±0.32**	**−1.55±0.59**
***speB***	**3.79±1.98**	**2.02±1.01**
***ska***	0.29±0.21	**−3.31±1.06**
***rofA***	**1.66±1.01**	**−**0.11±0.53
***eno***	0.39±0.19	0.54±0.28
***sagA***	**3.35±2.31**	**−**0.24±0.09
***lacA1***	**2.41±0.98**	**1.97±0.36**
***lacA2***	0.80±0.19	0.43±0.32
***fruK***	0.10±0.05	**−**0.18±0.06

Log_2_ expression ratios are shown. All genes with log values >1.0 are significantly induced, all genes with a negative log of<−1.0 are significantly reduced in the mutant strains.

## Discussion

In the present study we investigated the impact of the heterologous expression of the Ralp3 regulator on GAS M2 and M6 virulence characteristics to approach the question if the *ralp3* gene was deleted from the ERES region of those serotypes during evolution. In GAS the Ralp family regulators exist in four variants. RofA, also titled Ralp1, and Nra, also designated Ralp2, are encoded in a serotype-specific fashion within the FCT genomic region [12]. The GAS serotype M1 genome sequence [23] revealed the existence of two additional Ralp regulators, Ralp3 and Ralp4 [24]. Both were partially characterized [14], [17], [25]. Kreikemeyer and colleagues exclusively found *ralp3* homologues genes in the genomes of GAS serotypes M1, M4, M12, M28, and M49 [14]. Different studies showed that Ralp3 is a transcriptional regulator involved in GAS virulence and sugar utilization, but regulatory directions are apparently GAS serotype specific [14], [17], [25].

It is known that Ralp3 from GAS serotype M49 controls gene transcription from operons encoding sugar utilization enzymes. These include both *lac* operons and *fru* operon [17]. The *lac2* operon in GAS is involved in carbohydrate metabolism [26] and *lac1* encodes genes for virulence regulation [27]. In both tested sugars, fructose and lactose, no significant differences between M2/M6 wild type and *ralp3* expression strains were observed, if the strains were grown as single species. However, in direct competition experiments the *ralp3* free strains M2 and M6 showed growth advantages compared with the naturally *ralp3* expressing strain M49 in all tested media. All strains were inoculated at the same CFU. After entering the exponential growth phase, the M2 and M6 wild type strains utilized the tested sugars more efficiently, resulting in a significant outcompetition of the M49 wild type strain. If this phenotype is based on differences in sugar uptake/transport and/or metabolism needs to be determined in the future. However, these results let to the hypothesis that in GAS M2 and M6 the natural situation (lack of Ralp3 expression) infers a metabolic fitness superior to GAS M49. To challenge this hypothesis we created a heterologous Ralp3 expression mutant in the background of both the M2 and M6 wild type strains. Direct competition assays supported the hypothesis, as the same metabolic phenotype was observed during competition of M2/M6 wild type and M2::*ralp3*/M6::*ralp3* strains, respectively. Both wild type strains outcompeted the *ralp3* expressing strains at stationary growth phase in all tested media. The reason for this phenotype remains unclear and is currently under investigation in our lab by kinetic and bioinformatic models. However, these results gave first hints that Ralp3 expression in M2 and M6 would weaken the strains fitness, suggesting that the gene encoding Ralp3 was actively removed from the former ERES gene block in these strains.

GAS adherence to and invasion of host epithelial cells are essential early events in infection [3]. Our results show that heterologous expression of Ralp3 leads to a significantly decreased ability of GAS serotypes M2 and M6 to adhere to and internalize into the majority of tested epithelial cells. This again is another hint to why the Ralp3 encoding gene is missing in these strains. The qRT-PCR analysis supported these results. *mga* encodes one of the GAS virulence transcriptional regulators and *emm*, the major GAS adhesin as part of the Mga core-regulon, are down regulated in the M6::*ralp3* background. The role of the Mga regulon and M-Protein in GAS adherence and invasion is well characterized [2], [28]. The attenuated transcripts of these genes could lead to a limited amount of M-protein on the surface of GAS, and, as a final consequence result in reduced attachment and internalization to eukaryotic structures.

The changes in *emm* gene transcription are also important for survival in human media like blood or serum [2]. Reduced amounts of anti-phagocytic M-protein in both *ralp3* expressing strains could lead to a decreased protection against phagocytosis and impaired survival in human media.

Another observation in this study was the induced amount of SpeB in M2:*ralp3*/M6::*ralp3* supernatants. The induced amount of active SpeB on the surface of the bacteria is matched by an increased *speB* mRNA level. SpeB is a protease secreted by GAS and known to degrade a wide range of host and GAS proteins *in vitro*, especially M-protein, Protein H, Protein F1, Streptokinase, and C5a peptidase [29]. The increased amount of SpeB in supernatants of the M2:*ralp3*/M6::*ralp3* strains probably resulted in a degradation of the above mentioned factors, which in turn leads to a decreased ability of the mutants to survive in human media and to adhere on and internalize into human epithelial cells.

A further effect caused by increased amounts of SpeB, is the decreased amount of Ska especially in the supernatant of M6::*ralp3* strain. These data is consistent with the transcriptomic analysis. Ska, an important virulence factor of GAS, binds to inactive zymogen plasminogen, resulting in the production of active plasmin, the central protease of a fibrinolytic system [30]. Rezcallah and colleagues [31] showed that increased amounts of SpeB degrade Ska directly, which explains our observation.

In summary, we have shown that Ralp3 is a virulence regulator in a serotype specific fashion. In our experimental setup the heterologous expression of Ralp3 in GAS serotypes M2 and M6 leads to a significant impairment of virulence relevant characteristics of the mutants. Apparent from the data presented here is that GAS wild type strains have a differential metabolic fitness with exclusive importance in direct competition situations. To our knowledge this was not studied previously. Moreover, this could be another missing link for the explanation of GAS tissue tropism in a scenario where several strains arrive at the same tissue site, but metabolic fitness and adaptation of just one serotype leads to outcompetition of the others. At least in the M49 wild type Ralp3 expression is a fitness advantage, most likely explaining why this gene can be found in this serotype, In serotypes M2 and M6, two examples of ralp3-negative serotypes, the expression of Ralp3 leads to disadvantages in all tested phenotypes. The latter could be considered first evidence that the Ralp3-encoding gene was rather lost or actively deleted from the ERES gene block. However, at this time point we can not finally answer the question, if GAS serotypes have lost or gained the *ralp3* gene. Further experiments are needed and results have to be evaluated in future studies in order to acquire a better understanding of GAS pathogenisis in general, and serotype-specific occurrence of transcriptional regulators in particular.
